# Expeditious Generation of Biparatopic Common Light Chain Antibodies *via* Chicken Immunization and Yeast Display Screening

**DOI:** 10.3389/fimmu.2020.606878

**Published:** 2020-12-23

**Authors:** Jan P. Bogen, Stefania C. Carrara, David Fiebig, Julius Grzeschik, Björn Hock, Harald Kolmar

**Affiliations:** ^1^ Institute for Organic Chemistry and Biochemistry, Technical University of Darmstadt, Darmstadt, Germany; ^2^ Ferring Darmstadt Laboratory, Biologics Technology and Development, Darmstadt, Germany; ^3^ Ferring International Center S.A., Saint-Prex, Switzerland

**Keywords:** biparatopic antibody, antibody discovery, common light chain, yeast display, chicken-derived

## Abstract

Bispecific (BsAb) and biparatopic (BpAb) antibodies emerged as promising formats for therapeutic biologics exhibiting tailor-made functional properties. Over recent years, chicken-derived antibodies have gained traction for diagnostic and therapeutic applications due to their broad epitope coverage and convenience of library generation. Here we report the first generation of a biparatopic common light chain (cLC) chicken-derived antibody by an epitope binning-based screening approach using yeast surface display. The resulting monospecific antibodies target conformational epitopes on domain II or III of the epidermal growth factor receptor (EGFR) with lower double- or single-digit nanomolar affinities, respectively. Furthermore, the domain III targeting variant was shown to interfere with epidermal growth factor (EGF) binding. Utilizing the Knob-into-Hole technology (KiH), a biparatopic antibody with subnanomolar affinity was generated that facilitates clustering of soluble and cell-bound EGFR and displayed enhanced antibody-dependent cell-mediated cytotoxicity (ADCC) compared to the parental antibodies. This strategy for generating cLC-based biparatopic antibodies from immunized chickens may pave the way for their further development in therapeutic settings.

## Introduction

In recent years, antibody engineering aimed at generating next-generation antibody formats such as bispecific antibodies (bsAbs) gained massive interest since they can be programmed to possess multiple novel functionalities that cannot be mediated by conventional monoclonal antibodies (mAbs). BsAbs can simultaneously target two distinct antigens on different cells, thereby facilitating approximation of target and effector cells, leading to immunological effects that are unachievable with combinations of monospecific antibodies (1–5). Furthermore, simultaneous targeting of cancer-specific antigens and checkpoint inhibitors on the surface of the same malignant cell can elevate tumor-specific effects and contribute to the safety profile of a bispecific therapeutic antibody (6, 7). A particular subclass of BsAbs are biparatopic antibodies (BpAbs), which target two different epitopes on the same antigen.

The use of biparatopic antibodies in the frame of cancer cell targeting was exemplified by Li and co-workers, who generated a biparatopic anti-HER2 antibody-drug conjugate (ADC). The molecule facilitated enhanced receptor clustering, internalization, and lysosome trafficking compared to the classical monospecific format, resulting in degradation of the tumor (8). Recently, the Wang group published an anti-CD3 fragment antigen-binding (Fab) that was C-terminally fused to two camelid single-domain antibodies (VHHs) targeting non-overlapping epitopes on the cancer target HER2. This resulted in a trivalent, biparatopic construct showing potent T cell-mediated cytotoxicity *via* simultaneous CD3 and HER2 binding, even in low HER2-expressing cells (9). Even though such antibody fragments can mediate impressive cytotoxic effects, their usage *in vivo* is limited by their short half-life.

Recently, Akiba and co-workers described the formation of biparatopic antibody fragments based on a non-overlapping single-chain fragment variable (scFv) linked to either a SpyTag or a SpyCatcher. The subsequent formation of a covalent bond between the SpyCatcher and SpyTag creates a biparatopic molecule targeting two distinct epitopes on the cancer-related antigen roundabout homolog 1 (Robo1) (10). Although this technology is of major interest as a screening platform or for the generation of biosensors for diagnostic applications, the short half-life of these molecules and the lack of Fc-mediated effector functions lower their therapeutic potential.

**Figure 1:**
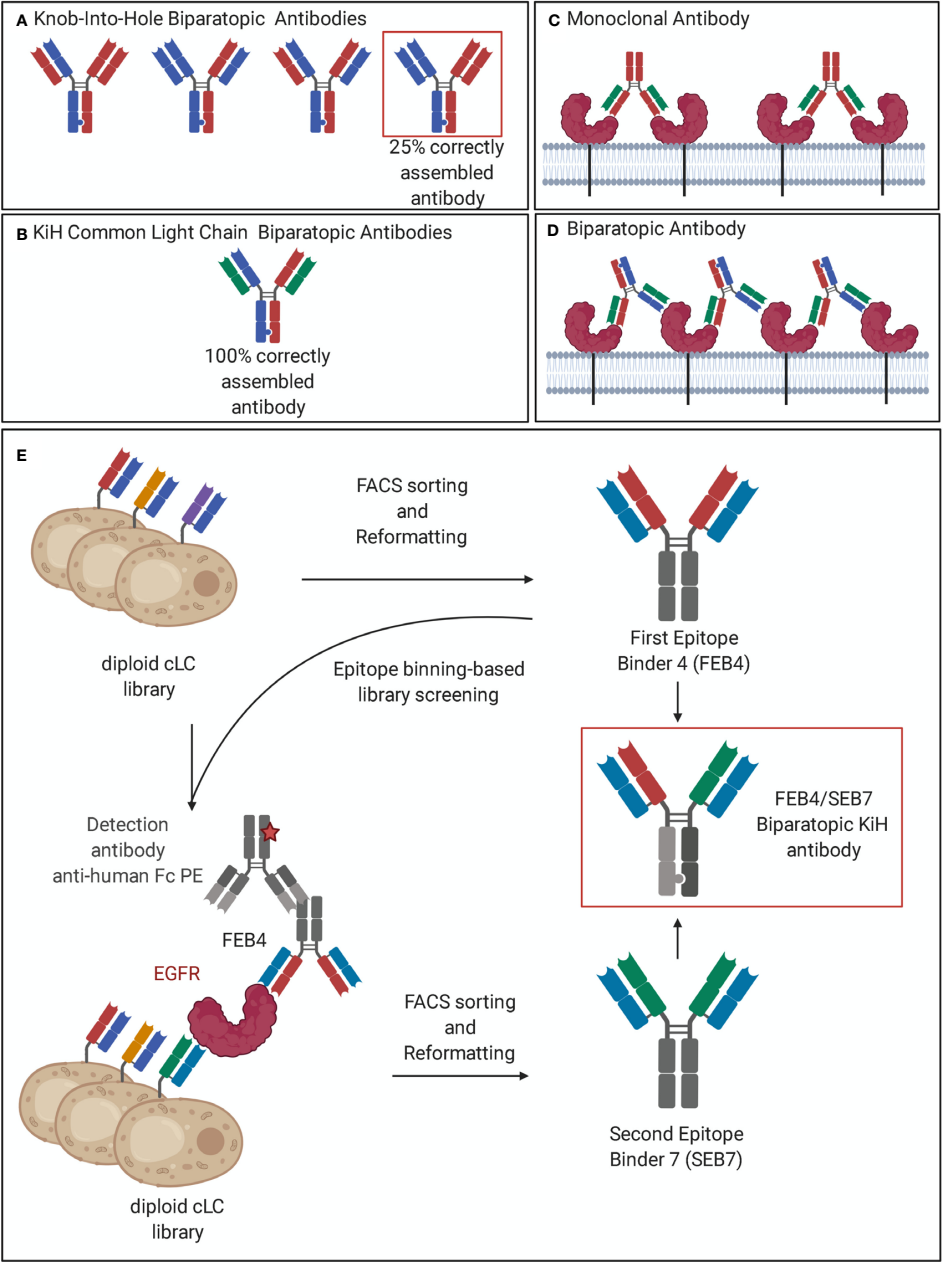
Common light chain biparatopic antibodies and schematic representation of the screening procedure. **(A)** The heterodimerization of the heavy chains is achieved by the Knob-into-Hole technology. Due to the need for correct pairing of heavy and light chains, only 25% of produced antibodies are correctly assembled. **(B)** The utilization of a common light chain pairing with both heavy chains enabled the circumvention of the light chain pairing problem. **(C)** Monoclonal antibodies recognize a single epitope of the target antigen and can therefore bind to two individual receptors. **(D)** Biparatopic antibodies bind to two distinct epitopes of the same antigen. By binding to two receptor molecules, additional epitopes on the target remain exposed allowing for the binding of an additional antibody, leading to crosslinking of the target receptor and receptor clustering. **(E)** Epitope binning-based FACS screening resulted in two VH domains, addressing orthogonal epitopes while comprising one common light chain. Subsequent reformatting into the Knob-into-Hole format enabled the production of a biparatopic antibody. Created with BioRender.com.

Over the last years, many different IgG-like bispecific formats have been investigated (11). Bispecific antibodies require two different binding arms. In the classical setting, antibodies containing two separate heavy chains are generated, that differ in the target binding ability of the corresponding VH domains. For the correct heterodimerization of the heavy chains, multiple platform technologies are available, such as Knob-into-hole (12), SEED-bodies (13), or the electrostatic steering approach (14), that all rely on preferred heterodimer formation of the two different Fc fragments. Nevertheless, even with perfect heavy chain pairing, the aforementioned technologies can only mediate the correct assembly of 25% of the produced antibody, since upon co-expression of two light chains, scrambled species may arise as a result of incorrect light chain paring (**Figure 1A**). While technologies like Cross-mab (15) or orthogonal Fab interfaces (16) are able to circumvent this problem, the utilization of a common light chain remains the most straightforward approach (17). In those BsAbs, a single light chain is used for pairing with both different heavy chains (**Figure 1B**). Notably, antigen binding is mainly mediated by the VH domains, while the common light chain provides stability rather than contributing to antigen binding (18). Obviously, this technology requires a straightforward strategy to isolate heavy-chain-only binders that do not rely on the contribution of the predefined light chain for target binding.

To date, common light chain antibodies have been generated for various bispecific applications (18–24). Nonetheless, they are mostly based on other formats, such as scFvs or camelid single-domain antibodies (VHH) (8–10, 25–29). Even though the generation of such molecules is straightforward, they do not exhibit an IgG-like architecture.

For decades, the most popular species for antibody generation upon immunization have been mice, rats, and rabbits. However, in many cases where the (human) target protein used for immunization has a high similarity with an ortholog present in the immunization host, a poor immune response is observed. The resulting antibodies might be limited in their epitope coverage due to the failure of the immune system of the host to recognize these epitopes as foreign (30). Based on their phylogenetic distance from humans, chicken immunization can result in antibodies against epitopes that are broadly conserved in mammal species and, therefore, not accessible by immunization of mammals (30–32). Furthermore, the avian gene diversification mechanism based on gene conversion (33–35), accounts for a fast and easy antibody library generation. No sequence variation occurs at the regions coding for the amino- and the carboxyterminal section of the VH and VL domains, thus allowing for the usage of non-degenerated primer pairs for gene amplification by PCR (36). Recently, our group established a fluorescence-activated cell sorting (FACS)-based yeast surface display (YSD) technology for the isolation of high affine chicken-derived antibodies (36–39). Additionally, we demonstrated the potential of incorporating a common light chain (cLC) within the screening procedure while maintaining affinity and a broad epitope coverage (40). In this study, we describe the isolation and characterization of the first biparatopic common light chain antibody targeting the extracellular domain of epidermal growth factor receptor (EGFR-ECD) (**Figures 1C, D**) that is derived from immunized chickens by combining a novel epitope binning-based screening strategy with the knob-into-hole technology for the generation of bispecific heavy chain pairs.

## Material and Methods

### Plasmids

For yeast surface display, pYD1-derived vectors (Yeast Display Vector Kit, version D, #V835-01, Thermo Fisher) were utilized, encoding either a tryptophan auxotrophic marker, an ampicillin resistance, and the aga2 signal peptide followed by the respective VH-CH1 encoding sequence, and the aga2 gene or a leucine auxotrophic marker, a kanamycin resistance gene and an αMFpp8 signal sequence followed by the VL-CL encoding sequence. Gene expression was controlled by the galactose-inducible promotor (GAL1). For the soluble expression of full-length chimeric antibodies, pTT5-derived vectors (40) were utilized, encoding either the heavy or the light chain constant domains. Biparatopic variants were expressed using pTT5-derived vectors encoding the full-length chimeric antibody with either a knob or hole mutation (12) within the CH3 sequence, and a C-terminal His- or Twin-StrepII-Tag, respectively. For one-armed antibodies, a pTT5-derived vector encoding the Hinge-CH2-CH3 with knob or hole mutations and C-terminal His- or Twin-StrepII-Tag were utilized.

### Yeast Strains, Libraries, and Sorting Procedure

The used yeast strains and their handling, as well as the utilized libraries, were described previously (40). Screening rounds were performed utilizing either a BD Influx FACS Cell sorter or a Sony SH800S. For the detection of surface presentation on chimeric Fabs, an anti-Kappa AF647 F(ab′)_2_ antibody (SouthernBiotech) was used. As antigen EGFR-ECD-Fc chimera (R&D) was utilized, and binding was detected *via* a goat anti-human IgG Fc PE-conjugated antibody (Thermo Fisher). For epitope binning-based sorting procedures, a monomeric EGFR-ECD molecule (produced in-house), as well as a chimeric full-length anti-EGFR mAb (FEB4), were utilized.

### Reformatting, Expression, and Purification of Chimeric Full-Length, One-Armed, or Biparatopic Antibodies

Plasmids were isolated from yeast cells using the Zymoprep Yeast Plasmid Miniprep kit (Zymoresearch) and were transformed into *E. coli* XL1-Blue, following the manufacturer’s instructions. Plasmids were isolated utilizing the Wizard^®^ Plus SV Miniprep Kit (Promega) and sequenced at Microsynth Seqlab (Göttingen). Resulting VH genes were amplified using Q5 polymerase (NEB), according to the manufacturer’s protocol, incorporating *SapI* sites and were subsequently subcloned into pTT5-derived vectors by Golden Gate cloning, as described previously (40). For soluble expression, Expi293F™ (Thermo Fisher, A14527) cells were transfected, harvested and secreted proteins purified by Protein A as described elsewhere (23). One-armed and biparatopic molecules were captured by IMAC (HisTrap HP, GE Healthcare), as described in (23), followed by Strep-Tactin XT affinity chromatography according to the manufacturer’s protocol. Buffer exchange against PBS was performed utilizing HiTrap^®^ Desalting columns (GE Healthcare).

### Epitope Binning and Mapping *via* Yeast Surface Display

YSD-based epitope binning was performed as described before (40). In brief, induced yeast cells, displaying either FEB4 or SEB7, were stained with 100 nM EGFR-ECD followed by incubation with either matuzumab, cetuximab, or PBS-B (PBS + 0.1% (w/v) BSA) as control. Antibody binding was verified by an anti-human Fc PE-conjugated antibody. YSD-based epitope mapping was performed as described before (41). In short, the ECD of EGFR was subdivided into segments consisting of residues 1-124, 1-176, 1-294, 273-621, 294-543, or 475-621, and displayed on yeast cells utilizing the pCT vector (42). The surface presentation was verified by a biotinylated anti-c-myc antibody (Miltenyi Biotech) and Streptavidin APC (Thermo Fisher). Subsequently, staining was performed using 200 nM of FEB4 or SEB7, followed by incubation with an anti-human Fc PE-conjugated antibody.

### Affinity Determination, Epitope Binning, Mixed-Site-Binding Assay and Epidermal Growth Factor Competition *via* Biolayer Interferometry

For affinity determination of chimeric antibodies, anti-human IgG Fc Capture (AHC) biosensors were soaked in PBS pH 7.4 for at least 10 min, and subsequently loaded with 10 µg/ml of the antibody of interest until a layer thickness of 0.7 to 1 nm was reached. Quenching and all subsequent steps were performed utilizing the kinetics buffer (FortéBio). Association was measured by using different concentrations of EGFR-ECD (produced in-house), ranging from 7.8 nM to 500 nM. As a negative control, kinetics buffer was used instead of antigen solution. Binding kinetics were determined based on Savitzky-Golay filtering and a 1:1 Langmuir binding model using the respective negative control.

Epitope binning was performed in a tandem setup with EGFR-ECD (10 µg/ml) immobilized on Ni-NTA tips with a threshold of 0.7 nm. Subsequently, 400 nM of the first antibody was applied for 900 s, followed by either the same or the second antibody at the same concentration and time. Increment of layer thickness in the last step is an indicator of non-overlapping epitopes.

For the determination of a clean- or mixed-site binding, the biparatopic molecule was biotinylated utilizing EZ-Link™ Sulfo-NHS-LC-Biotin (Thermo Fisher) according to the manufacturer’s protocol. 10 µg/ml was immobilized on SAX-biosensor tips until saturation was reached. Subsequently, either 250 nM EGFR-ECD or buffer was applied for 600 s, followed by an association step of the non-modified biparatopic antibody. An increment of layer thickness compared between the EGFR-ECD and the buffer sample verifies a mixed-site binding.

For the EGF competition assay, the antibodies were loaded onto AHC tips with 10 µg/ml until a layer thickness of 0.7 nm was reached. Subsequently, 100 nM EGFR-ECD preincubated with either 0 nM, 100 nM or 1000 nM EGF was applied for 600 s.

The Octet RED96 system (FortéBio, Molecular Devices) was utilized for all measurements at 30°C and 1000 rpm.

### Nano DSF and Size Exclusion Chromatography

Thermal stability measurements and SEC profiles were determined as previously described (40).

### Cultivation of A431 Cells

A431 human epidermoid carcinoma cells (ATCC^®^ CRL-1555™) were cultured in Dulbecco’s Modified Eagle Medium (DMEM, Thermo Fisher), supplemented with 10% fetal bovine serum (FBS) superior (Merck Millipore), and 1% Penicillin-Streptomycin (Sigma Aldrich P0781). The cells were cultivated in T75 cell culture flasks at 37°C in a humidified atmosphere with 5% CO_2_ and passaged every 3 to 4 days after reaching approximately 80% confluency.

### Antibody-Dependent Cell-Mediated Cytotoxicity

ADCC assays were performed using the Promega ADCC Reporter Bioassay Kit (G7010) following the manufacturer’s instructions. A fourfold serial dilution of respective antibodies (5 µg/ml to 305 pg/ml) was incubated with 12.500 A431 cells and effector cells. After 6 h of incubation at 37°C and 5% CO_2_, luciferase activity was measured and plotted against the antibody concentration. For the fitting, a variable slope four-parameter fit was utilized.

## Results

### Identification of VH Domains Targeting Orthogonal Epitopes

For the generation of a biparatopic antibody, two different antigen binders are required that share the same common light chain but recognize different epitopes on the target protein. As a model antigen for the isolation of biparatopic chicken-derived antibodies, the extracellular domains of the human epidermal growth factor receptor (EGFR-ECD) were used. Various monoclonal antibodies against this target exist, including the therapeutic antibodies cetuximab and matuzumab (43, 44). To obtain anti-EGFR common light chain antibodies, a chicken was immunized with EGFR-ECD as described (37), and the VH encoding genes were amplified and transferred into the yeast expression vector pYD1 (40). Subsequently, this VH library was paired with a single common light chain (termed H2) that was derived from a chicken antibody directed against human chorionic gonadotropin (hCG) (37, 40) by yeast mating. This light chain was chosen since it displayed favorable physicochemical features, such as high stability and low aggregation when combined with various heavy chains (40).

5×10^8^ clones of the resulting library were screened for three rounds *via* FACS using decreasing antigen concentrations starting from 250 nM down to 1 nM, to assure the enrichment of high affine antibodies (**Supplementary Figure 1A**). The resulting single clones were analyzed by flow cytometry, with the clone FEB4 (First Epitope Binder 4) showing the strongest binding to EGFR (**Supplementary Figure 1B**). Titration of target antigen on yeast cells showed a strong binding even at low molecular concentrations (**Supplementary Figure 1C**). The VH sequence (**Supplementary Figure 1D**) was reformatted into a chimeric IgG1 with a human Fc fragment format utilizing Golden Gate subcloning (40). Subsequently, FEB4 was transiently produced in Expi293F™ cells and purified using Protein A affinity chromatography.

To generate a biparatopic antibody against EGFR-ECD, another VH domain targeting an orthogonal epitope had to be isolated. To achieve this, an epitope binning-based screening approach was conducted (**Figure 1E**). This was based on incubating the yeast library with the target protein, His-tagged EGFR-ECD, followed by the addition of FEB4 being detected with a fluorescently labeled anti-human Fc antibody. Only when different epitopes are recognized, binding of FEB4 and cell staining can be expected to occur (**Supplementary Figure 2A**).

At first, the library after the 2^nd^ round was stained utilizing 100 nM of EGFR-Fc chimera, revealing that 15.1% of the displayed Fab fragments within the library were able to bind EGFR (**Supplementary Figure 2B**). The yeast library after the 2^nd^ round was stained for the epitope-binning-based screening using either 100 nM or 10 nM monomeric His-tagged EGFR-ECD (produced in-house) respectively, followed by incubation with 200 nM of the chimeric FEB4-IgG1 antibody. FEB4 binding was detected utilizing a goat anti-human IgG Fc secondary PE-conjugated antibody. At 100 nM EGFR-His, a significant reduction of the cell-binding population was observed, indicating the presence of antibodies within the library targeting overlapping epitopes with FEB4 (**Supplementary Figure 2B**). Nevertheless, a significant proportion showed target binding (7.95%). At 10 nM EGFR-His concentration, only 2.45% of yeast cells displayed a Fab targeting an orthogonal epitope to FEB4 (**Supplementary Figure 2B**). To assure the selection of high-affinity binders, this cell population was sorted. Ten resulting clones were randomly chosen and analyzed by flow cytometry for target binding in an epitope-binning-based manner (**Supplementary Figure 2C**). The variant SEB7 (Second Epitope Binder 7) showed the strongest binding signal when binding was verified utilizing FEB4, indicating an affine antibody targeting an orthogonal epitope to FEB4. SEB7 (**Supplementary Figure 2D**) was subsequently reformatted using the Golden Gate strategy into a chimeric IgG1 antibody and transiently expressed using Expi293F™ cells, followed by Protein A purification.

### Light Chain Effects on Binding Properties

To investigate the influence of the cLC on the binding behavior, the VL domains of H1 and H3, derived from previously reported chicken-derived antibodies (37), were paired with the VH domains of FEB4 and SEB7, respectively, using YSD. These VL sequences differ in CDR-lengths and amino acid compositions (**Supplementary Figure 3A**). Induced yeast clones expressing respective antibodies were stained utilizing 100, 10, and 1 nM EGFR-Fc chimera. While FEB4 showed similar binding behavior independent of the used light chain in a dose-dependent manner, SEB7 displayed significant antigen recognition only when paired with the H2 light chain (**Supplementary Figure 3B**). These results indicated that the VL domain only stabilizes the FEB4-Fab but is not involved in antigen binding, while in SEB7, the H2 cLC is mandatory to facilitate EGFR binding.

### Epitope Binning and Epitope Mapping

Epitopes targeted by FEB4 and SEB7 were characterized by YSD-based epitope binning using a sandwich setup (**Supplementary Figure 4**). FEB4 competed with the binding of the anti-EGFR antibody matuzumab to EGFR but did not interfere with cetuximab binding. SEB7 did not interfere with the binding of either matuzumab or cetuximab.

**Figure 2:**
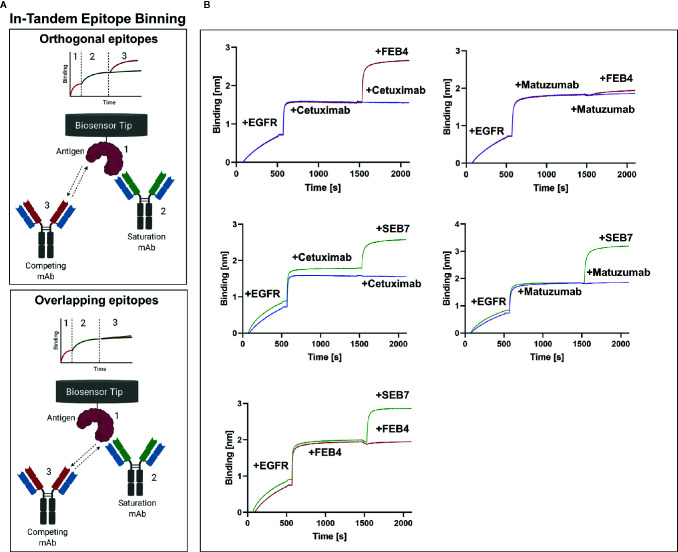
Epitope binning of FEB4 and SEB7. **(A)** Schematic representation of epitope binning in-tandem setup. His-tagged antigen is loaded onto Ni-NTA biosensors (1), followed by binding of the first antibody until saturation is achieved (2). Subsequently, either the saturation antibody or the completion antibody is applied (3), resulting only in an increment of layer thickness if orthogonal epitopes are addressed. **(B)** BLI-based binning in-tandem setup investigating epitope recognition of FEB4 and SEB7. Ni-NTA tips were loaded with EGFR-ECD, followed by binding to the first antibody of interest until saturation was achieved. Subsequently, either the second antibody was applied, or a new incubation of the first antibody was performed, which served as control. Created with BioRender.com.

Additionally, biolayer interferometry (BLI)-assisted epitope binning in the in-tandem setup was performed (**Figure 2A**). In accordance with YSD-based measurements, BLI measurements corroborate the notion that FEB4 shares an overlapping epitope with matuzumab but not cetuximab. SEB7, on the other hand, neither targets the cetuximab nor the matuzumab epitope. Furthermore, it was shown that FEB4 and SEB7 target orthogonal epitopes (**Figure 2B**), proving the successful application of epitope binning in sandwich setup within the antibody screening process.

**Figure 3:**
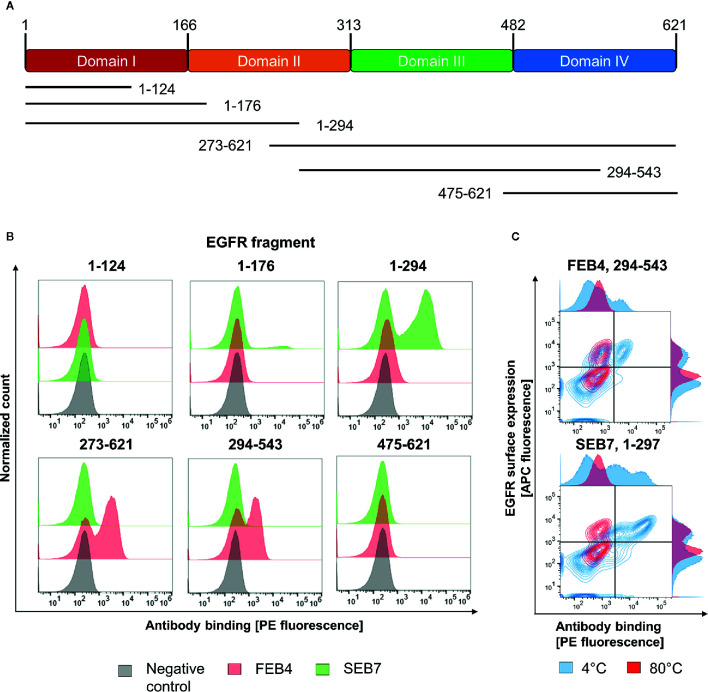
Epitope analysis of FEB4 and SEB7. **(A)** Schematic representation of EGFR domains and the fragments investigated in this study. **(B)** YSD-based epitope mapping. Binding of FEB4 and SEB7 to yeast cells expressing different EGFR-derived domains were detected utilizing the goat anti-human Fc PE antibody. **(C)** Conformational epitope recognition on various truncated versions of EGFR-ECD. Yeast cells expressing the targeted EGFR-derived fragments were either incubated at 4°C or 80°C, respectively, for 30 min. Surface presentation was verified by the anti-c-myc biotin antibody and Streptavidin APC, binding of FEB4 and SEB7 was measured utilizing the anti-human Fc PE antibody.

While the epitope of FEB4 could be localized to the matuzumab binding site, localization of the SEB7 epitope remained unclear. To investigate which of the four domains of EGFR-ECD is targeted by SEB7, flow cytometry analysis was performed using yeast cells displaying truncated versions of the EGFR extracellular domains (41) (**Figure 3A**). Display of EGFR segments was verified by detection of the C-terminal c-myc tag (data not shown). Incubation of induced yeast cells with 200 nM of the respective antibody, followed by subsequent staining using the goat anti-human Fc PE antibody, allowed for domain-level epitope mapping (**Figure 3B**).

Since FEB4 targets the EGFR fragments 273-621 and 294-543, it was mapped to EGFR domain III, which is consistent with the prior YSD- and BLI-based binning experiments. SEB7 showed strong binding to fragment 1-297 and marginal, but specific, binding toward segment 1-176, allowing mapping to EGFR domain II.

To investigate whether FEB4 and SEB7 target linear or conformational epitopes, yeast cells displaying the fragment 1-297 or 294-543 were incubated either at 80°C or 4°C, respectively, as described before (41). Subsequently, yeast cells were stained with either FEB4 or SEB7. Heat-incubated yeast cells were still positive for c-myc presentation, indicating that the corresponding EGFR segment was still presented at full-length. Nevertheless, the binding of FEB4 and SEB7 to their respective fragments was abolished entirely due to heat-mediated protein denaturation, indicating that both antibodies target conformation-specific epitopes (**Figure 3C**). A previous report by Cochran and co-workers demonstrated that these EGFR fragments do not undergo refolding when displayed on yeast cells (41).

### Construction of a Biparatopic Antibody

To ensure the heterodimerization of heavy chains, the Knob-into-Hole technology was utilized (12). The VH domain of FEB4 was subcloned into the coding sequence of a CH1-Knob-Fc, C-terminally fused to a Twin-StrepII-tag, while SEB7 was subcloned into a CH1-Hole-Fc comprising a C-terminal His-tag. C-terminal tags allowed for the specific purification of correctly heterodimerized KiH antibodies and the difference in size enabled a straightforward analysis *via* SDS-PAGE. Both variants were transiently produced as one-armed (oa) variants comprising their respective KiH counterparts with a lacking Fab region or as a full-length biparatopic antibody in Expi293F™ cells.

Besides the often observed hole-hole dimers, expression of KiH antibodies also results in knob-knob homodimers, albeit to a lower extent (45, 46). To purify only correctly assembled bpAbs, the first purification was performed *via* immobilized metal affinity chromatography (IMAC) to deplete knob-knob dimers and any additional proteins from the cell culture supernatant. To purify only correctly heterodimerized antibodies, a subsequent Strep-Tactin XT chromatography was performed.

SDS-PAGE analysis revealed Twin-StrepII-tagged Fcs showing a significantly higher molecular weight than His-tagged Hole-Fcs, allowing the distinguishment between both chains (**Supplementary Figure 5**). All chains exhibited comparable staining intensities, indicating the purification of correctly heterodimerized antibodies.

### Biophysical Characterization

Parental antibodies FEB4 and SEB7 were analyzed utilizing BLI, resulting in notable affinities of 29.3 nM and 1.14 nM, respectively (**Supplementary Figure 6**; **Table 1**). One-armed variants of both antibodies exhibited comparable affinities and association and dissociation rates. In comparison, FEB4 exhibited a higher association rate but also a higher off-rate, while SEB7 exhibited a lower on- but also lower off-rate. The BpAb combined the high on-rate with a lower off-rate resulting in an improved affinity of 637 pM upon EGFR-ECD binding compared to the parental antibodies.

**Table 1:** Biophysical properties of FEB4 and SEB7 variants including affinity, kinetic binding rates, melting temperature, and aggregation.

Antibody	K_D_ [nM]	k_on_ [M^−1^ s^−1^]	k_dis_ [s^−1^]	TM [°C]	Aggregation [%]
FEB4	29.3 ± 0.579	5.19 × 10^5^ ± 9.36×10^3^	1.52×10^−2^ ± 1.24×10^−4^	65.2	1.65
oaFEB4	31.2 ± 1.53	5.05 × 10^5^ ± 2.35×10^4^	1.58×10^−2^ ± 2.40×10^−4^	67.8	10.45
SEB7	1.14 ± 0.149	4.28 × 10^5^ ± 5.21×10^3^	4.87×10^−4^ ± 6.33×10^−5^	67.6	0.00
oaSEB7	1.84 ± 0.624	2.51 × 10^5^ ± 1.56×10^4^	4.62×10^−4^ ± 1.54×10^−4^	67.8	10.25
FEB4/SEB7	0.637 ± 0.00637	4.54 × 10^5^ ± 2.45×10^3^	2.89×10^−4^ ± 2.74×10^−6^	66.8	6.60

The thermostabilities of all generated antibodies were measured utilizing the NanoDSF technology, resulting in TM values between 65.2°C and 67.8°C, indicating high thermal stability of all variants (**Supplementary Figure 7A**). In full-length antibodies, two to three TM values are expected, referring to unfolding of the Fab fragment and the CH2/CH3 domains (47). However, as an antibody is only functional if all domains are folded correctly, the lowest TM value was utilized to compare the stability of all generated antibodies. Additionally, size-exclusion chromatography (SEC) profiles demonstrated that the parental antibodies exhibit favorable properties with nearly no measurable aggregation (**Supplementary Figure 7B**). Knob-into-Hole variants showed 6.60% aggregates and revealed expected retention times (**Supplementary Figure 7B**, **Table 1**).

### Epidermal Growth Factor Competition

**Figure 4:**
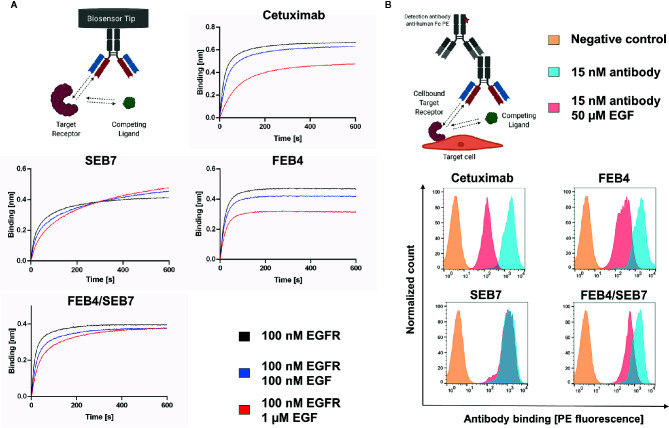
EGF competition assays. **(A)** BLI-based EGF competition. Using AHC biosensors, the depicted antibodies were loaded followed by association to EGFR preincubated with varying EGF concentrations. **(B)** EGF-dependent binding to EGFR^+++^ A431 cells. 1×10^6^ A431 cells were stained utilizing antibodies preincubated with or without EGF. Binding to A431 cells was measured by FACS using the goat anti-human Fc PE antibody. Created with BioRender.com.

Binding of epidermal growth factor triggers conformational changes of the EGF receptor and facilitates receptor dimerization, leading to downstream signaling (48). To verify whether FEB4/SEB7 and its parental antibodies interfere with the EGF binding ability of EGFR, EGF competition assays were conducted. As described above, our data indicate that FEB4 targets EGFR domain III and has an overlapping epitope with matuzumab, hinting that it might target an epitope proximate or overlapping to the EGF ligand binding site. BLI analysis was performed with antibodies loaded onto AHC biosensor tips and binding to EGFR preincubated with EGF at different concentrations was measured. These experiments revealed EGF inhibited both cetuximab and FEB4 from binding to EGFR in a dose-dependent manner, while SEB7 bound to the receptor complex independently of the applied EGF concentration (**Figure 4A**). Compared to FEB4, the BpAb showed a stronger binding towards EGFR at higher EGF concentrations, comparable with observed association curves of SEB7. Nevertheless, the BpAb did not reach the layer thickness of the EGF-negative control similar to FEB4 and cetuximab, indicating that FEB4/SEB7 combines EGF-dependent and independent binding.

These results were verified by flow cytometric analysis of cellular-binding to EGFR-overexpressing A431 cells, in the presence of a high concentration of EGF (**Figure 4B**). While all antibodies showed strong cell binding, cetuximab and FEB4 showed significantly impaired cell binding in the presence of EGF. SEB7 bound A431 cells independently of the EGF concentration, underlining its EGF-independent binding behavior. The BpAb showed intermediate EGF-dependence, caused by its ability to bind in an EGF-dependent and -independent manner.

### Clustering Assay

**Figure 5:**
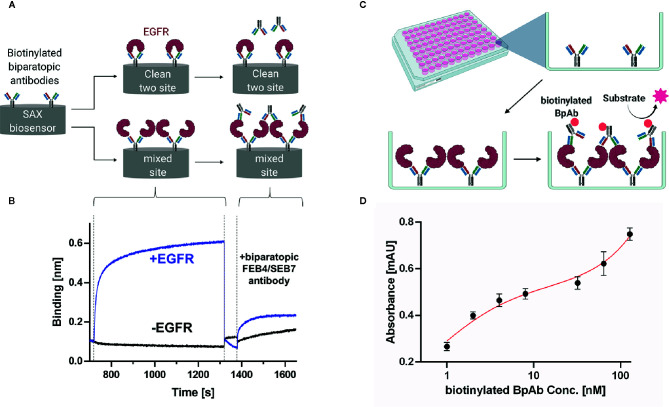
Clustering assay of the biparatopic antibody. **(A)** Schematic representation of BLI-based two-site binding assay that can either result in a clean two-site binding or a mixed site binding. **(B)** BLI-based two-site binding assay. Biotinylated BpAb was loaded onto SAX tips followed by incubation with EGFR-ECD or kinetics buffer, respectively. Subsequently, binding of unmodified BpAb to the complex was analyzed. Each step was aligned separately **(C)** Schematic representation of ELISA-based two-site binding assay. **(D)** ELISA of BpAb binned against itself. 50 µl of a 25-nM BpAb solution was coated on a 96-well MaxiSorp MTP (ThermoFisher) overnight, followed by incubation with 200 nM EGFR-ECD and varying concentrations of biotinylated BpAb (1–128 nM). Binding was detected utilizing Streptavidin-HRP and TMB-One solution (Promega). Each concentration was measured in triplicates and analyzed using GraphPad Prism 8. A two-site specific binding fit was applied. Created with BioRender.com.

Biparatopic antibodies can bind to two different epitopes on the target antigen. Those interaction events can occur either in a 1:1 (clean site binding) or a 2:1 (mixed site binding) antibody-antigen ratio (**Figure 5A**). In the latter case, the antigen can cluster on the surface of the targeted cell, resulting in different biological outcomes. We decided to investigate whether the biparatopic FEB4/SEB7 antibody facilitates the clustering of EGFR molecules. To this end, binning of the BpAb against itself was performed in a BLI-based assay. Biotinylated FEB4/SEB7 was loaded onto SAX tips, followed by association of EGFR. Incubation with unmodified FEB4/SEB7 resulted in an increased layer thickness, absent in the sample control lacking EGFR. This indicated that one EGFR molecule could be bound simultaneously by two different BpAbs in a mixed site binding manner (**Figure 5B**).

Additionally, an ELISA-based clustering assay was conducted where the BpAb was coated, followed by incubation with EGFR-ECD and subsequently with biotinylated biparatopic antibody (**Figure 5C**). Detection *via* streptavidin conjugated to horseradish peroxidase (HRP) (Thermo Fisher) led to a biphasic dose-dependent binding curve, indicating biparatopic and mixed-site binding (**Figure 5D**).

Furthermore, EGFR clustering was verified by SEC, where FEB4/SEB7 and its respective parental antibodies were preincubated with a 1.2-fold molar excess of EGFR (**Supplementary Figure 8**). While FEB4 and SEB7 bound EGFR as observed by lower retention times, the parental species remained detectable at higher retention times. FEB4/SEB7 showed a mass shift to a significantly shorter retention time, indicating a higher degree in clustering caused by the biparatopic binding behavior. Additionally, the peak of the solitary BpAb nearly completely vanished, demonstrating nearly quantitative binding.

### Antibody-Dependent Cell-Mediated Cytotoxicity

**Figure 6:**
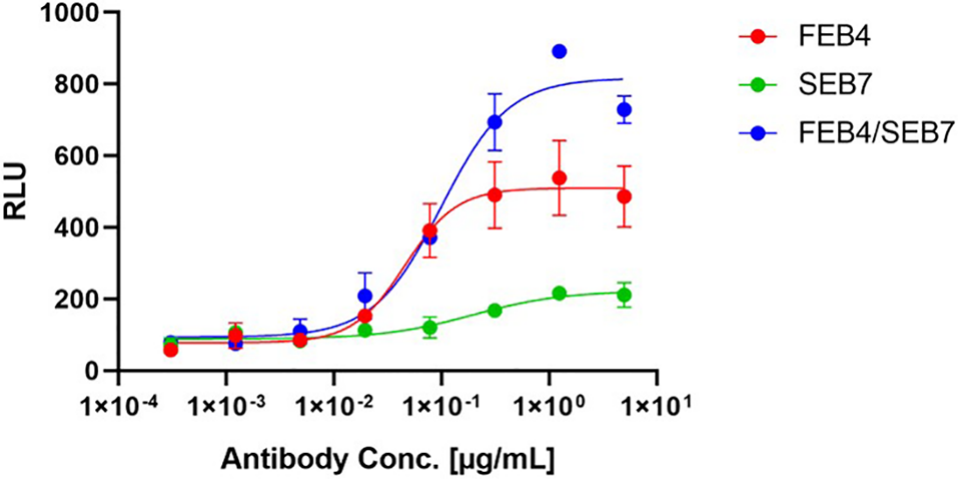
ADCC Assay of parental and biparatopic antibodies on A431 cells. The Promega ADCC assay was performed utilizing the parental and the biparatopic variants. 12,500 A431 cells were cultured overnight before incubation with a fourfold serial dilution of antibodies (5 µg/ml to 305 pg/ml) and ADCC effector cells for 6 h. Luciferase signal is plotted against the logarithmic antibody concentration. EC_50_ values: FEB4, 45.8 pM; SEB7, 191.5 pM; FEB4/SEB7, 98.9 pM.

Biparatopic antibodies are able to cluster their respective receptors, which in return can lead to a high local presentation of Fc parts on the surface of the target cell. NK cells expressing CD16 can bind those clustered Fc parts, leading to an enhanced antibody-dependent cell-mediated cytotoxicity (ADCC). To evaluate whether the biparatopic FEB4/SEB7 construct could facilitate such a mode of action, an ADCC assay was performed that relies on NFAT activation upon CD16 clustering, leading to the expression of luciferase. The biparatopic antibody-mediated a significantly stronger fold induction compared to the parental variants (**Figure 6**). FEB4 outperformed SEB7 regarding EC_50_ value and fold induction, indicating that ADCC and effector cell engagement are independent of affinity (**Table 1**). These results indicate a favorable effector cell engagement and activation mediated by the biparatopic construct.

## Discussion

In 2020, two biparatopic antibody candidates have been under clinical investigation for the treatment of breast cancer: Zanidatamab (ZW25) in phase II studies (NCT numbers: NCT04224272, NCT04276493, NCT03929666) and the ZW25-derived antibody-drug conjugate ZW49 in a phase I study (NCT number: NCT03821233). These asymmetric antibodies comprise a heterodimerized Fc, which either carries a scFv targeting HER2 domain IV or a Fab targeting HER2 domain II (49). Even though a large number of asymmetric and symmetric biparatopic antibodies exist in literature and preclinical research, most do not exhibit an IgG-like architecture (8–10, 25–29).

In this study, we generated the first biparatopic cLC antibody resulting in an IgG-like molecule. To this end, we isolated one EGFR-specific antibody in a conventional FACS screening approach using YSD. The resulting mAb termed FEB4 was utilized in a novel epitope binning-based screening approach to isolate a second antibody termed SEB7. Both antibodies target orthogonal epitopes and comprise the H2 common light chain. The latter facilitates a favorable aggregation behavior and notable thermostabilities, consistent with previously published results (40). The presented epitope-binning based screening procedure on cells allows for the rapid isolation of antibodies exhibiting non-overlapping epitopes and bypasses the need for extensive binning experiments.

By flow cytometric cell-binding assays and biolayer interferometric measurements, it was shown that FEB4 is able to bind EGFR in an EGF-dependent manner. FEB4 targets an epitope on the domain III of EGFR, which together with domain I, mediates the binding to EGF (50). Cetuximab is known to bind domain III and inhibit EGF binding (51). Even though FEB4 exhibits an orthogonal epitope to cetuximab, it has an overlapping binding site with matuzumab, which also binds to domain III and inhibits the dimerization of EGFR molecules upon conformational changes after EGF binding (52). It is therefore tempting to speculate that FEB4 might be able to inhibit EGF binding and receptor dimerization. Since SEB7 binds to domain II, no EGF dependence was expected.

SEB7 exhibits a nearly 30-fold higher affinity towards EGFR compared to FEB4, which originates from the high off rate of the latter. The epitope binning-based screening might favor the isolation of antibodies with higher affinities, since the detection antibody’s affinity is limiting. The difference in affinity and kinetic binding rates might be responsible for the observed mixed site binding behavior. While SEB7 binds tightly to its epitope, FEB4 can dissociate from the target molecule and bind to a second receptor. Its continuous binding and release might lead to stronger clustering than a BpAb that binds tightly with both Fab arms.

Our data indicate that the BpAb facilitates receptor clustering of soluble and cell-bound EGFR. Crosslinking of EGFR mediated by the biparatopic antibody and the concomitant clustering of the IgG1 Fc eventually mediated the activation of clustered CD16 expressed on effector cells, leading to a robust ADCC effect. Even though the EC_50_ values of FEB4 and the biparatopic construct are comparable, the higher fold induction of the bpAb indicates a stronger effector cell activation.

This clustering is likely due to the mixed site binding behavior of the biparatopic antibody, where the FEB4 arm binds to domain III on one EGFR molecule, and the SEB7 arm binds domain II on a second molecule. Due to this mixed site binding, the antibody is most probably not able to “chelate” its antigen, resulting in a relatively small increment of affinity (53). Nevertheless, this binding mode results in an increased clustering effect. On the other hand, a clean-site binding, where a single BpAb binds both epitopes on the same antigen molecule, leads to a more potent “chelating” effect resulting in a notable increase of affinity (53, 54). However, the chelation of one antigen by one BpAb would probably not result in elevated receptor clustering.

Regardless of the intended mode of action, this type of BsAb is a chimera that contains chicken-derived VL and VH domains, transplanted onto a human IgG1 scaffold. For potential therapeutic applications, humanization of the chicken-derived part of the molecule is mandatory. The utilization of a human VL domain as a cLC could ease the process of subsequent humanization. In OmniChicken, transgene chickens exhibiting human antibody germline sequences, the IGLV1-44 germline is implemented (30). As this human germline-encoded cLC shares approx. 44% homology to the avian H2 VL domain (data not shown), a pairing with a chicken-derived VH would most probably not result in a functional antibody. Our group recently demonstrated a fast and straightforward solution to humanize avian-derived antibodies *via* CDR loop transplantation onto human scaffolds, randomization of key residues that dictate loop orientation followed by functional screening using yeast surface display (55). Experiments leading to humanized bispecific and biparatopic chicken-derived antibodies are currently in progress.

Taken together, we present a straightforward method for the isolation of biparatopic antibodies, generated by an epitope binning-based YSD screening. The resulting antibodies target orthogonal and conformational epitopes on domain II and III of EGFR-ECD with notable affinities while exhibiting favorable biophysical properties and aggregation behavior. The derived biparatopic antibody is able to facilitate receptor clustering and combines EGF-dependent and -independent binding characteristics of its parental variants. To our knowledge, this represents the first common light chain-based biparatopic antibody. Furthermore, our work paves the way for clinical applications of chicken-derived antibodies and new engineering strategies for biparatopic antibodies in general.

## Data Availability Statement

The original contributions presented in the study are included in the article/**Supplementary Material**; further inquiries can be directed to the corresponding author.

## Author Contributions

JB and HK conceived and designed the experiments. JB and SC performed the experiments. JB, SC, DF, JG, and HK analyzed the data. DF, JG, and BH gave scientific advice. JB and HK wrote the paper. All authors contributed to the article and approved the submitted version.

## Funding

This work was supported by the Ferring Darmstadt Labs at Technical University of Darmstadt and by the department of GPRD at Ferring Holding S.A., Saint-Prex. The funders had no role in study design, data collection, and analysis, decision to publish, or preparation of the manuscript.

## Conflict of Interest

JG and BH were employed by the company Ferring Pharmaceuticals. JB, SC, and DF are employed by TU Darmstadt in frame of a collaboration project with Ferring Pharmaceuticals.

The remaining authors declare that the research was conducted in the absence of any commercial or financial relationships that could be construed as a potential conflict of interest.
