# Supplementary material for: Expeditious Generation of Biparatopic Common Light Chain Antibodies via Chicken Immunization and Yeast Display Screening
Source: Front Immunol. 2020 Dec 23;11:606878. doi: 10.3389/fimmu.2020.606878 (PMC7786285; doi:10.3389/fimmu.2020.606878)
Supplement: Supplementary file 1 [file DataSheet_1.pdf]

Supplementary Material

Supplementary Figures

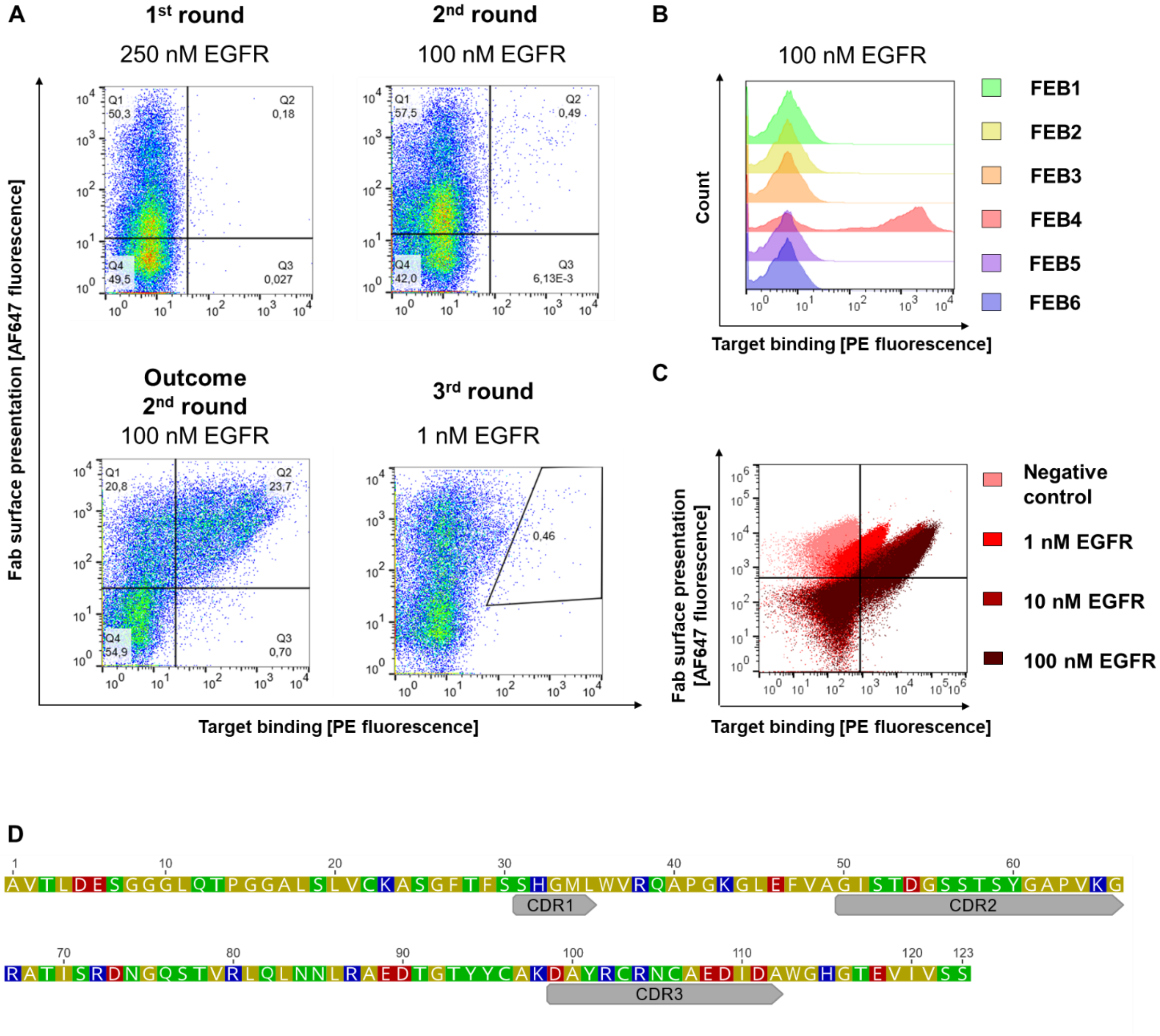

Supplementary Figure 1. Isolation of FEB4. (A) The screening of the YSD library comprising H2 common light chains was performed by FACS. Surface presentation was verified using an anti-kappa goat F(ab′)$_2$ antibody conjugated with AlexaFluor647. Target binding to EGFR-Fc chimera (R&D) was detected by utilization of an anti-human Fc PE-conjugated antibody. The percentage of cells per gate is depicted. (B) Six single clones were verified for EGFR binding. Solely FEB4 showed significant binding to EGFR and was further characterized. (C) Titration of EGFR-Fc chimera on FEB4-expressing yeast cells indicating strong binding at low antigen concentrations. (D) VH sequence of FEB4, the CDRs are shown.

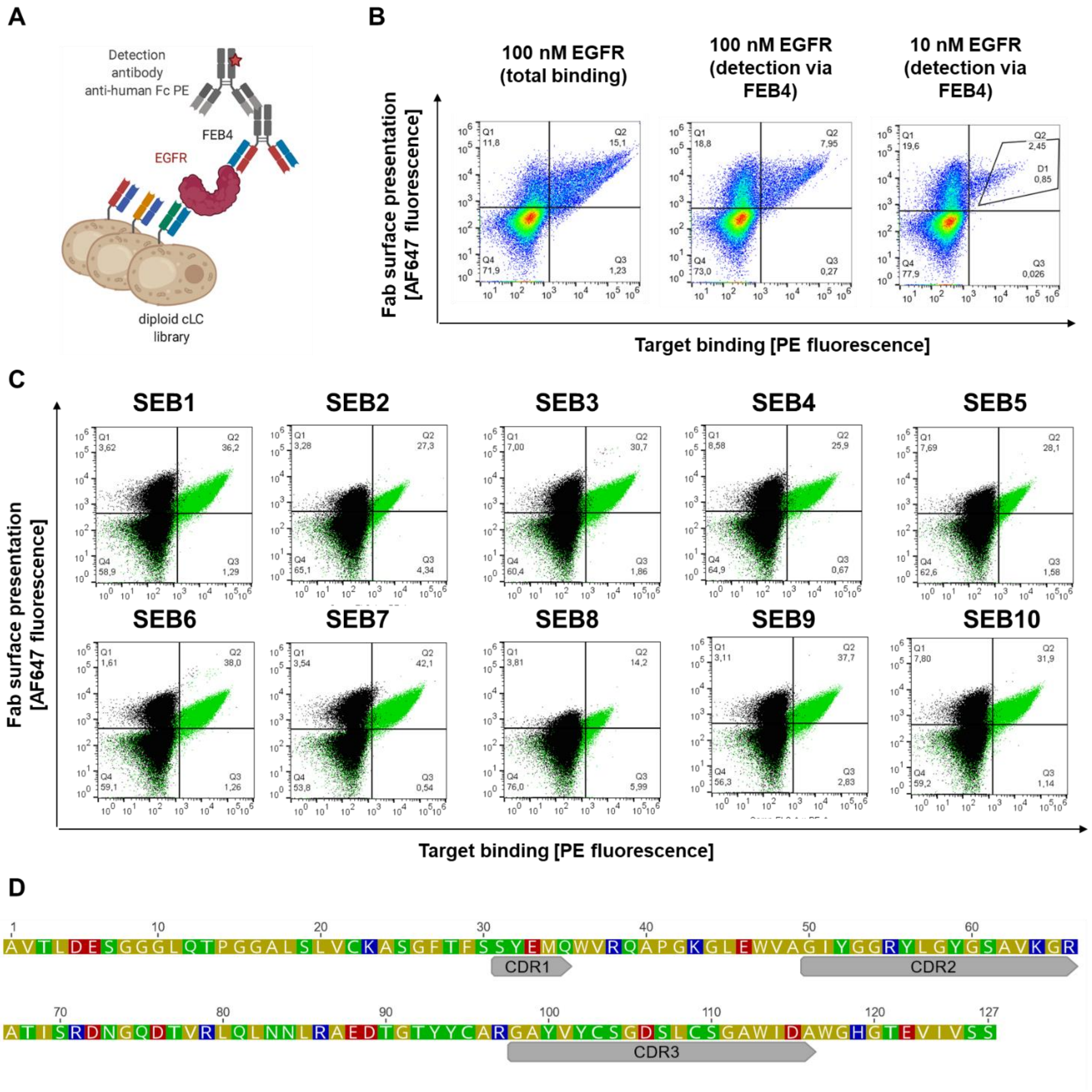

Supplementary Figure 2. Epitope binning-based screening and isolation of SEB7. (A) Schematic representation of an epitope-binning-based screening approach. (B) After the 2nd round (Fig. S2), the library was either stained with EGFR-Fc chimera (total binding) or monomeric EGFR-ECD followed by incubation with FEB4. Binding was verified by an anti-human Fc PE secondary antibody. Surface presentation was verified by an anti-kappa AF647-conjugated antibody. (C) Single clone analysis of second epitope binders using the binning-based staining procedure, with SEB7 showing the strongest binding signal. (D) VH sequence of SEB7, the CDRs are indicated. Created with BioRender.com.

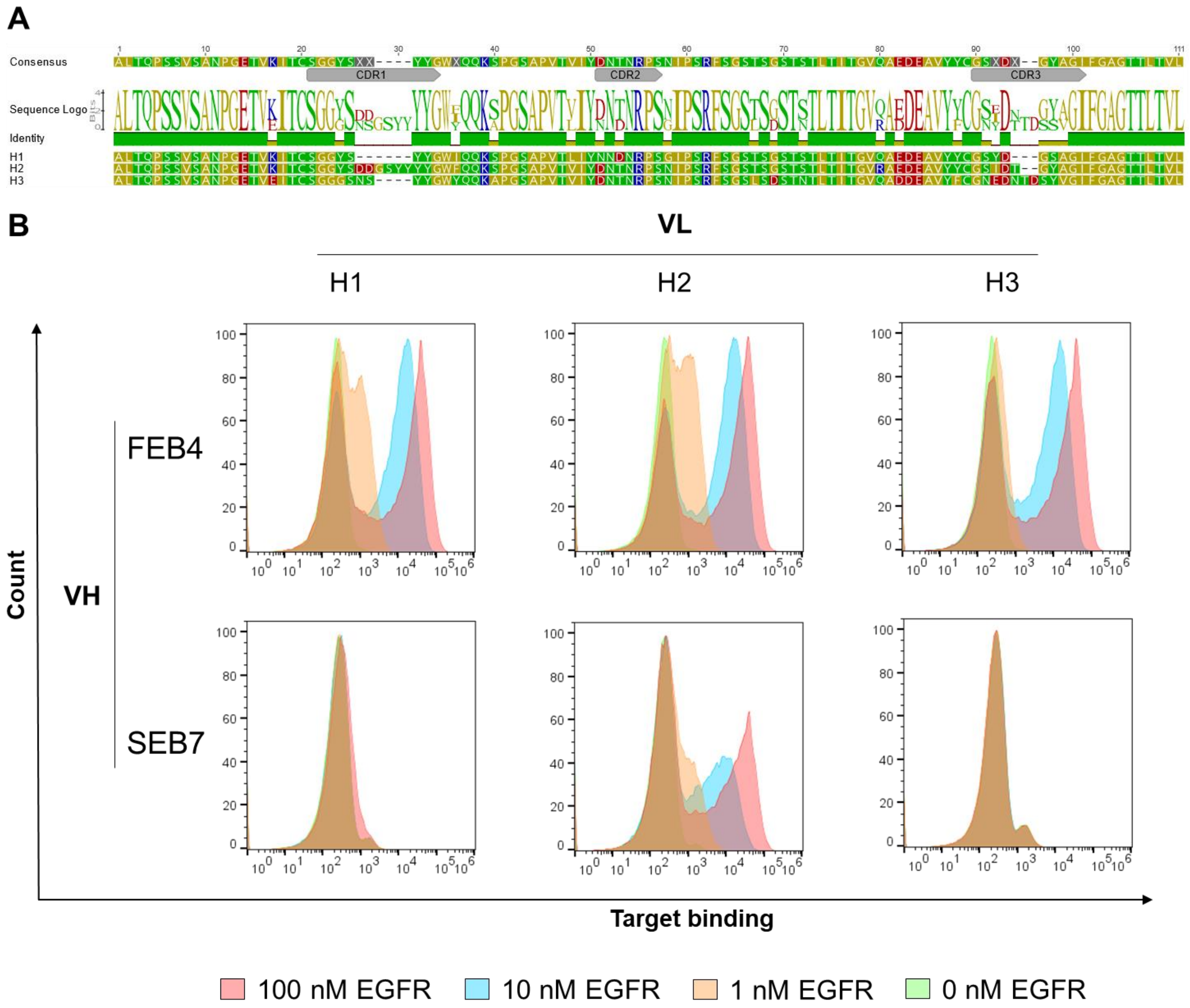

Supplementary Figure 3. Exchange of VL domains in FEB4 and SEB7 Fab-fragments. (A) The amino acid sequences of the H1, H2, and H3 VL are depicted. (B) YSD of FEB4- and SEB7-derived Fabs, exhibiting one of the three light chains. Following the incubation with either 100 nM, 10 nM, 1 nM, or 0 nM EGFR-Fc, target binding was verified utilizing an anti-human Fc PE secondary antibody. Cell counts are plotted against target binding fluorescence intensities.

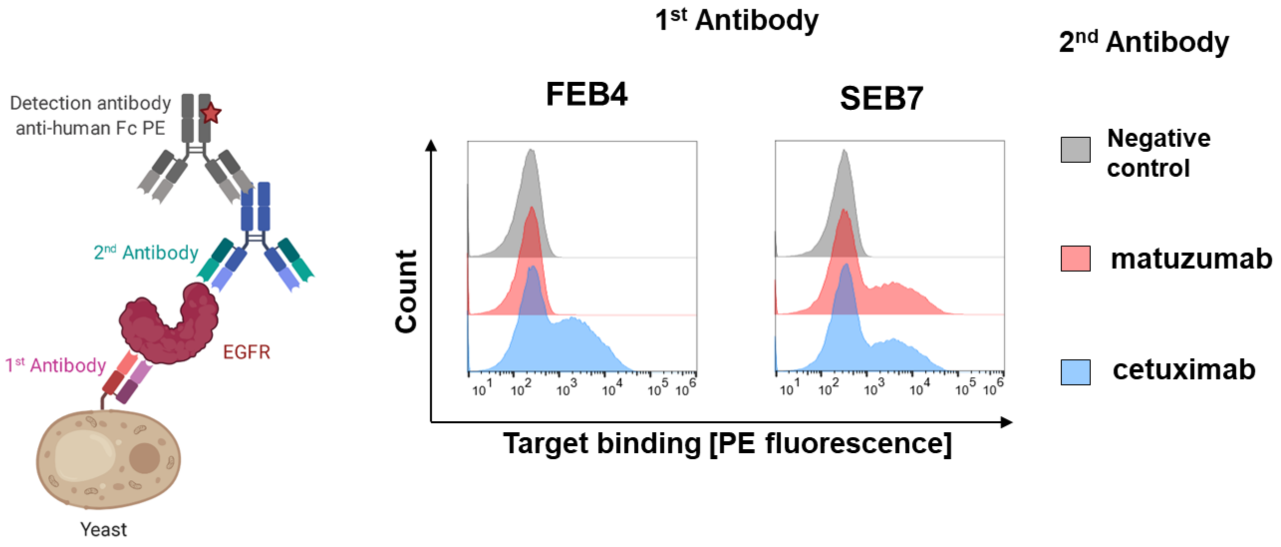

Supplementary Figure 4. YSD-based binning in sandwich setup. FEB4 or SEB7 were displayed on the yeast surface and stained with monovalent EGFR-ECD. Following the incubation of either matuzumab and cetuximab, binding was verified by anti-human Fc PE secondary antibody. Created with BioRender.com.

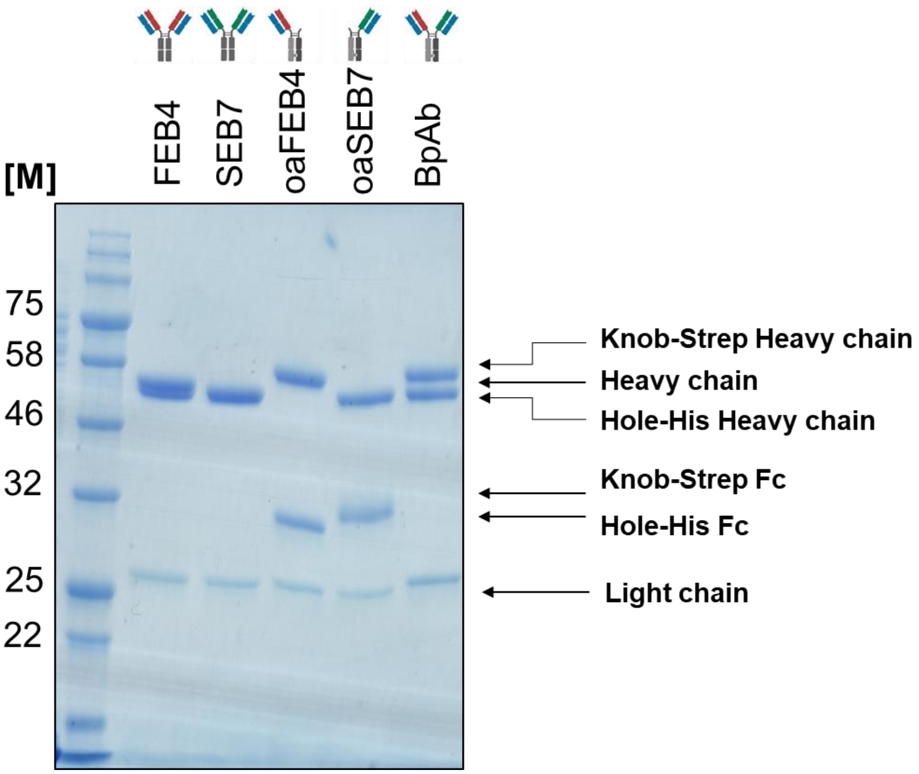

Supplementary Figure 5. SDS PAGE of mono- and biparatopic antibody variants. Different chain heavy chain sizes due to additional tags are indicated. Created with BioRender.com.

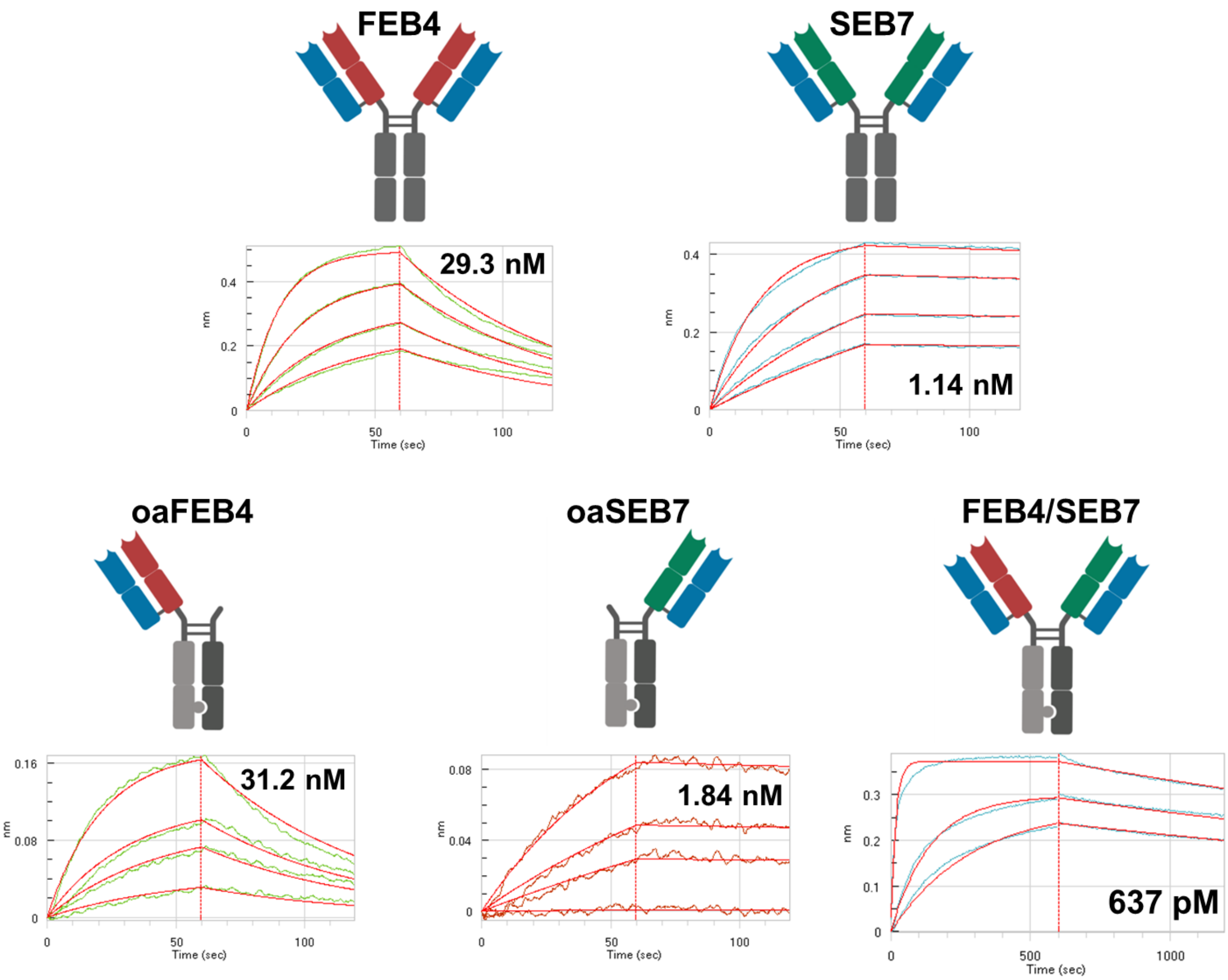

Supplementary Figure 6. Affinity determination of FEB4 and SEB7 as full-length, one-armed, and biparatopic variants, respectively. AHC tips were loaded with the antibody of interest followed by association and dissociation of EGFR-ECD. Binding curves were fitted utilizing the 1:1 binding model with Savitzky-Golay filtering. Created with BioRender.com.

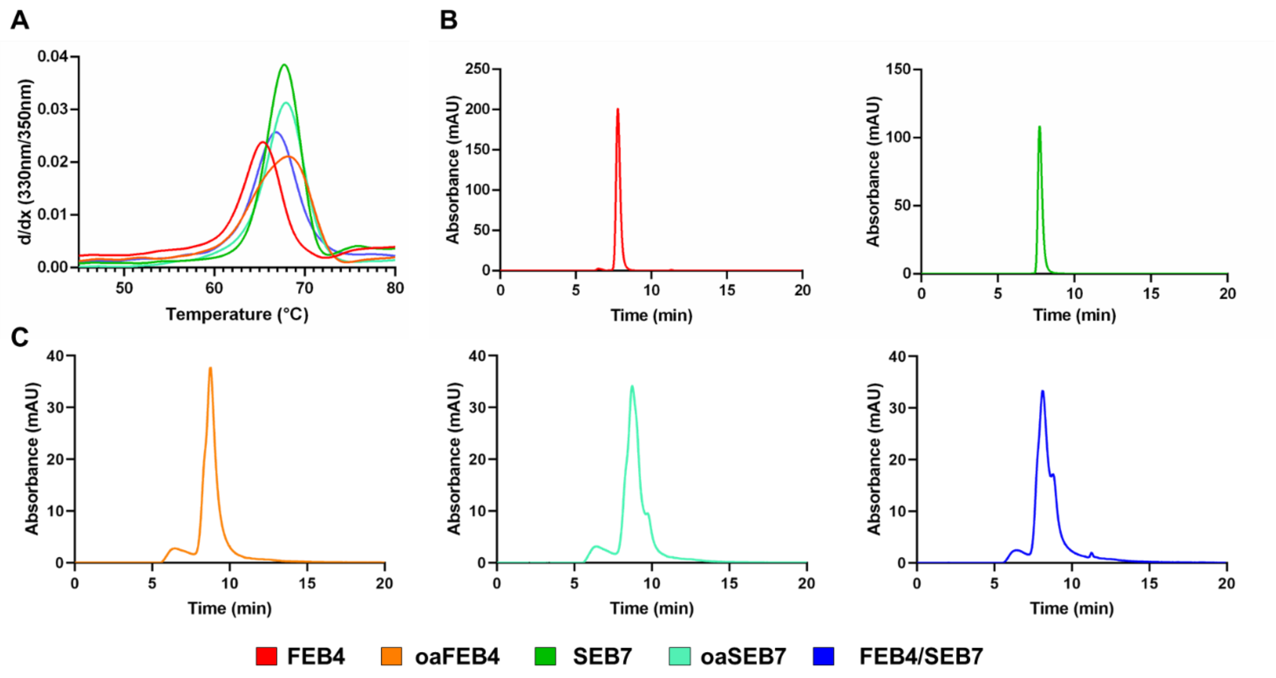

Supplementary Figure 7. Thermal stability and aggregation profiles. (A) The thermal stability was analyzed using the Prometheus NT.48 nanoDSF device (NanoTemper Technologies GmbH). The ratio of integrated fluorescence at 350 nm/330 nm was calculated at a heating rate of 1 °C/min. (B) SEC profiles of all FEB4 and SEB7 variants. A TSKgel SuperSW3000 column (Tosoh Bioscience) at a flow rate of 0.35 mL/min was utilized with PBS as mobile phase. 22 µL of a 0.5 mg/mL antibody solution was applied, and protein elution was detected at 280 nm.

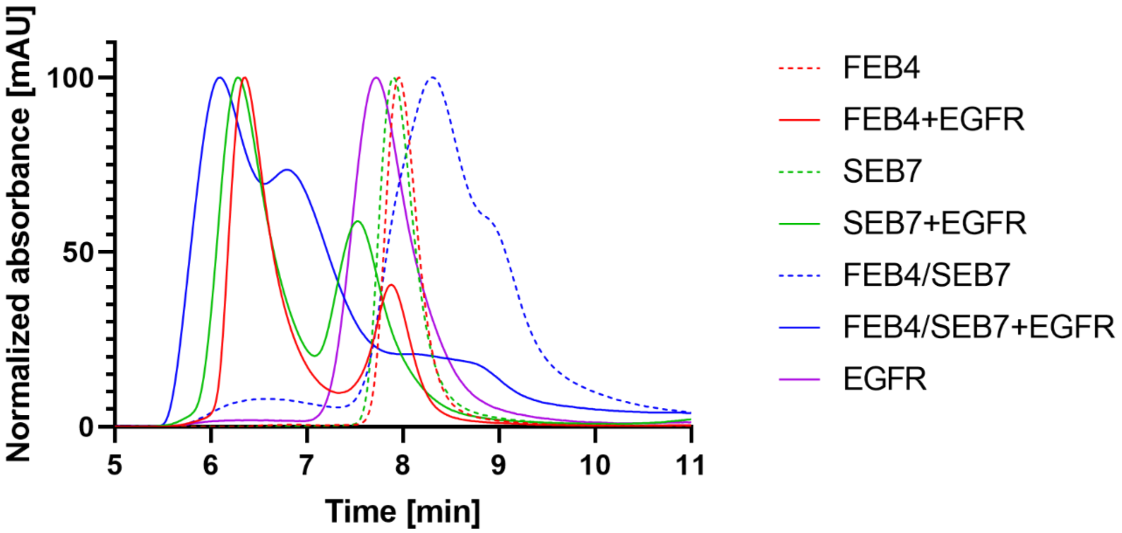

Supplementary Figure 8. SEC-based clustering Assay. A 0.5 mg/mL antibody solution was preincubated with a 1.2-fold molar excess of EGFR-ECD, and 22 µl were applied onto a TSKgel SuperSW3000 column (Tosoh Bioscience) at a flow rate of 0.35 mL/min. PBS was used as mobile phase. EGFR-ECD and solitary antibodies were analyzed as controls.
